# Emerging mechanisms of the unfolded protein response in therapeutic resistance: from chemotherapy to Immunotherapy

**DOI:** 10.1186/s12964-023-01438-0

**Published:** 2024-01-31

**Authors:** Jiang He, You Zhou, Lunquan Sun

**Affiliations:** 1 Xiangya Cancer Center, Xiangya Hospital, Central South University, Changsha, 410008 China; 2 Key Laboratory of Molecular Radiation Oncology Hunan Province, Changsha, 410008 China; 3 National Clinical Research Center for Geriatric Disorders, Xiangya Hospital, Changsha, 410008 Huan China; 4 Hunan International Science and Technology Collaboration Base of Precision Medicine for Cancer, Changsha, 410008 China; 5 Center for Molecular Imaging of Central, South University, Xiangya Hospital, Changsha, 410008 China; 6 Department of Pathology, Tongji Medical College Union Hospital, Huazhong University of Science and Technology, Wuhan, 430022 China

**Keywords:** Unfolded protein response, T-cell exhaustion, Immune checkpoint therapy, Chemotherapy

## Abstract

The accumulation of unfolded or misfolded proteins in the endoplasmic reticulum (ER) causes ER stress and activates the unfolded protein response (UPR). As an adaptive cellular response to hostile microenvironments, such as hypoxia, nutrient deprivation, oxidative stress, and chemotherapeutic drugs, the UPR is activated in diverse cancer types and functions as a dynamic tumour promoter in cancer development; this role of the UPR indicates that regulation of the UPR can be utilized as a target for tumour treatment. T-cell exhaustion mainly refers to effector T cells losing their effector functions and expressing inhibitory receptors, leading to tumour immune evasion and the loss of tumour control. Emerging evidence suggests that the UPR plays a crucial role in T-cell exhaustion, immune evasion, and resistance to immunotherapy. In this review, we summarize the molecular basis of UPR activation, the effect of the UPR on immune evasion, the emerging mechanisms of the UPR in chemotherapy and immunotherapy resistance, and agents that target the UPR for tumour therapeutics. An understanding of the role of the UPR in immune evasion and therapeutic resistance will be helpful to identify new therapeutic modalities for cancer treatment.

Video Abstract

**Supplementary Information:**

The online version contains supplementary material available at 10.1186/s12964-023-01438-0.

## Introduction

The endoplasmic reticulum (ER) is an extensive membrane-bound organelle in eukaryotic cells that plays an important role in many cellular processes, such as the storage and release of calcium [1]. Ample evidence suggests that nutrient fluctuations, hypoxia and pathological insults result in the accumulation of unfolded or misfolded proteins in the ER lumen and disruption of protein-folding homeostasis, a condition referred to as ER stress [1–3]. In response to ER stress, cytoprotective signalling pathways are triggered to restore protein-folding homeostasis in the ER in a process termed the unfolded protein response (UPR). When the UPR is triggered, global protein synthesis is transiently attenuated, and protein degradation pathways, including ER-associated degradation (ERAD) and autophagy, are activated, thus restoring ER homeostasis. If the protein-folding defect is persistent, cells undergo apoptosis. Thus, chronic or persistent ER stress results in cellular injury which is associated with various human diseases, including neurodegeneration, diabetes and cancers [2, 4, 5]. Chemotherapy is an additional extrinsic challenge that leads to the activation of UPR signalling. Previous studies have shown that the UPR plays an important role in chemotherapy resistance [6–8], suggesting that targeting the UPR is a promising strategy for tumour treatment. Understanding the mechanism of UPR-mediated resistance to antitumour drugs could provide a reference for developing a new therapeutic approach to overcome chemotherapy resistance.

Immunosuppression is crucial for tumorigenesis and tumour development, metastasis and recurrence. Establishing an immunosuppressive network derived from interactions between cancer cells and host stromal cells (e.g., T cells, macrophages, B cells, fibroblasts, dendritic cells) in the tumour microenvironment could promote tumour growth, protect the tumour from immune attack, and attenuate the efficacy of immunotherapeutic approaches [9–11]. Tumour-infiltrating T cells in the tumour microenvironment often lose their ability to kill tumour cells and are in a state of exhaustion. Exhausted T cells have the following two characteristics: (1) high expression of immune checkpoint molecules under persistent infection or antigen stimulation, such as programmed cell death protein 1 (PD1) [12–14], lymphocyte activating 3 (LAG3) [15], cytotoxic T-lymphocyte associated protein 4 (CTLA4) [16], T-cell immunoreceptor with Ig and ITIM domains (TIGIT) [17], CD160 [18], TNF receptor superfamily member 9 (TNFRSF9) [19], and hepatitis A virus cellular receptor 2 (HAVCR2) [20]; and (2) decreased proliferation, viability, and the loss of cytotoxic activity against tumours in vitro [21]. Although T-cell exhaustion does not effectively control tumour cells, this process is reversible. The clinical application of immune checkpoint therapies (ICTs), including anti-PD1/PD-L1 (PD1 ligand 1) antibody therapy, can restore T-cell immune function to a certain extent and increased the likelihood of tumour patient survival [22]. Overwhelming evidence indicates that the combination of PD1 with HAVCR2, CTLA4 or TIGIT inhibitors can more effectively improve T-cell function and eliminate tumour cells [23]. An increasing number of studies have shown that the UPR plays an important role in resistance to immune checkpoint inhibitors (ICIs). Thus, targeting the UPR may be a promising therapeutic strategy for overcoming resistance to ICIs.

In this article, we reviewed the molecular basis of UPR activation, the effect of the UPR on T-cell exhaustion and immune evasion, mechanisms of the UPR in resistance to chemotherapy and immunotherapy, and agents that target the UPR for tumour therapeutics. Finally, we discuss the applications and limitations of targeting the UPR in cancer treatment.

## Key sensors in the UPR

**Fig. 1:**
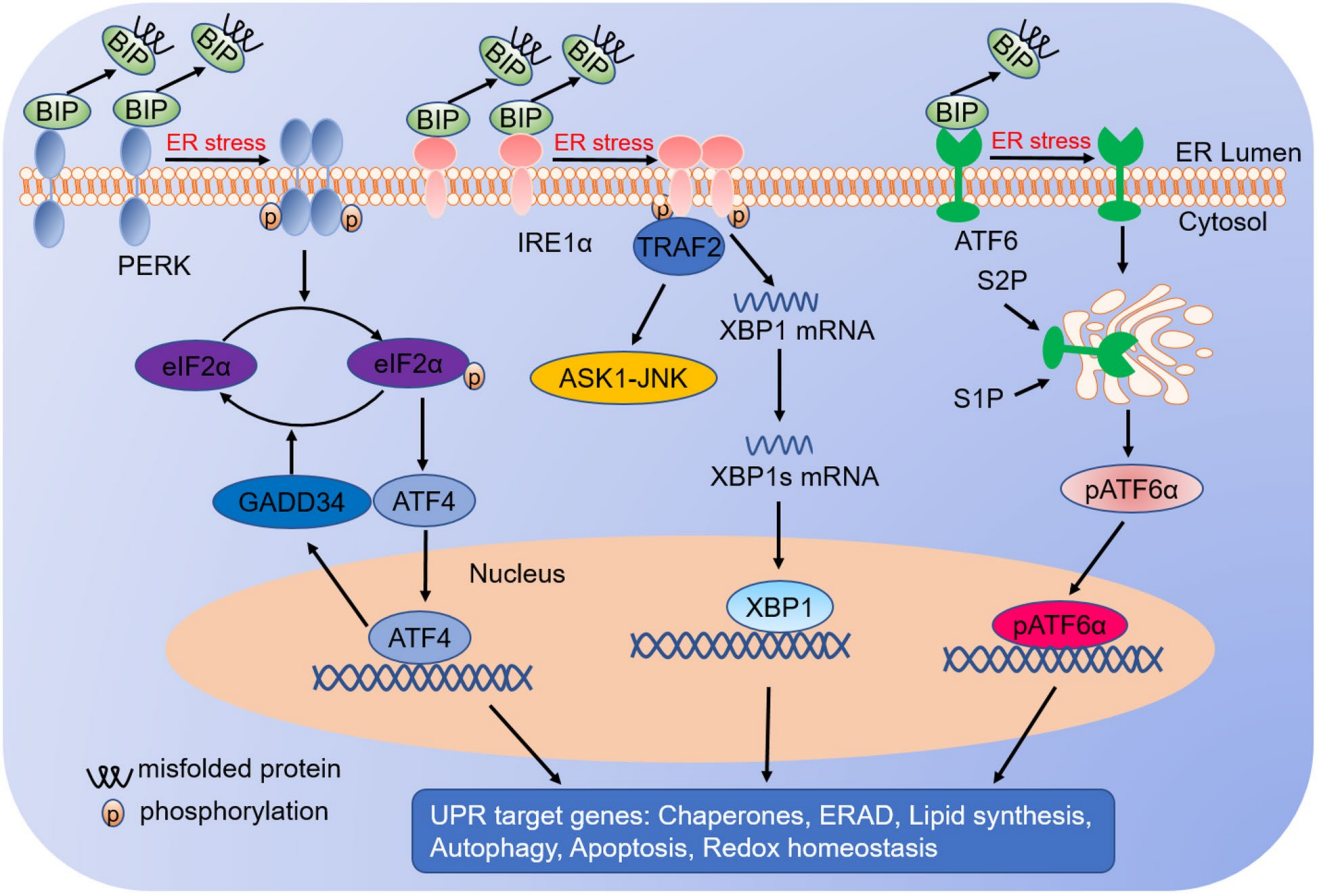
Unfolded protein response. Three ER stress sensors (PERK, IRE1α and ATF6α) are present in the ER membrane. Accumulation of misfolded protein in the ER lumen causes ER stress and dissociation of BiP from UPR sensors. Subsequently, IRE1α and PERK auto-transphosphorylated and are activated, and ATF6α exposes an ER export motif and translocates to the nucleus. Activated PERK phosphorylates eIF2α and leads to attenuation of global translation, but increases the expression of transcription factor ATF4. ATF4 induces transcription of genes that restore proteostasis and promote survival. Once ER stress is resolved, the dephosphorylation of eIF2α is triggered by growth arrest and DNA damage-inducible protein 34 (GADD34), and translation is reinitiated. Activated IRE1α has endoribonuclease activity and can splice an intron from unspliced X-box binding protein 1 (XBP1u), which leads to the production of XBP1s mRNA. Subsequently, XBP1s mRNA increases transcription of a set of genes associated with protein folding and degradation of misfolded proteins. Meanwhile, ATF6α is translocated from ER to Golgi, in which it is processed by S1P and S2P, producing a cytosolic p50 fragment (pATF6α) that functions as a transcription factor. Collectively, UPR activation aims to restore ER homeostasis, otherwise cells enter death

In mammals, UPR signalling is mainly transduced by the following three sensors: protein kinase R-like endoplasmic reticulum kinase (PERK) [24], activating transcription factor 6 alpha (ATF6α) [25], and inositol requiring enzyme 1 alpha (IRE1α) [26]. PERK and IRE1α are type I transmembrane proteins with cytosolic kinase domains. Under nonstress conditions, binding-immunoglobulin protein (BIP; also known as GRP78) binds to the luminal domain of PERK, IRE1α and ATF6α to prevent their activation. When unfolded and misfolded proteins accumulate in the ER lumen, BIP dissociates from PERK, IRE1α and ATF6α and binds to unfolded proteins, which allows PERK and IRE1α homodimerization and autophosphorylation [27, 28] and ATF6α translocation to the Golgi apparatus [29] (Fig. 1). A recent study showed that the above sensors could also be activated without disrupting their binding with BIP [30], which is indicative of the complexity of UPR signalling.

Under ER stress, PERK phosphorylates eIF2α, a translation initiation factor 2 subunit, leading to inactivation of eIF2α and inhibition of global protein translation due to the limited amount of the eIF2α–GTP–tRNA met ternary complex [31–33]. Although the translation of most mRNAs is suppressed in response to ER stress, the translation of some species of mRNA is increased, such as transcription factor ATF4 and C/EBP homologous protein (CHOP; also known as DDIT3). ATF4 is a transcription factor that regulates the expression of genes associated with the ER stress response, the antioxidant response, amino acid biosynthesis, autophagy and apoptosis [34–36] (Fig. 1). ATF4 also upregulates the transcription of CHOP and the growth of arrest and DNA damage-inducible protein 34 (GADD34; also known as PPP1R15A) [37, 38] (Fig. 1). CHOP is a pro-apoptotic factor that upregulates the expression of apoptosis-related genes, such as DR5 and PUMA [39–41]. In addition, PERK also phosphorylates and activates nuclear factor erythroid 2-related factor 2 (NRF2) to upregulate the expression of antioxidant genes, such as glutathione synthetase and haem oxygenase-1 (HO-1), thereby limiting the accumulation of cellular reactive oxygen species (ROS) [42–44].

IRE1α, a type I transmembrane protein, contains a kinase domain and an endoribonuclease (RNase) domain at its cytoplasmic tail [45]. In response to ER stress, IRE1α dimerizes and transautophosphorylates, inducing conformational alteration of its RNase domain and thereby activating its RNase activity. Afterwards, IRE1α splices a 26-nucleotide intron from the XBP1 mRNA and produces XBP1s mRNA, leading to a shift of the reading frame to translate a stable and active transcription factor known as XBP1s [1, 46–49] (Fig. 1). XBP1s upregulates a subset of target genes associated with lipid synthesis, protein folding, secretion, protein degradation, redox homeostasis, and amino acid metabolism [50, 51] (Fig. 1), which helps cells restore ER homeostasis. In addition, IRE1α increases the expression of several genes associated with cell death in response to ER stress. For instance, IRE1α utilizes its RNase activity to selectively cleave microRNA that normally suppresses the expression of pro-apoptosis targets, such as pro-oxidant protein TXNIP (thioredoxin-interacting protein) and caspase-2, resulting in increased expression of these pro-apoptosis genes [52–54]. IRE1α also interacts with the adaptor protein tumour necrosis factor (TNF) receptor-associated factor 2 (TRAF2) to recruit apoptosis signal-regulating kinase 1 (ASK1) and JUN N-terminal kinase (JNK), leading to activation of the apoptosis pathway [55–57] (Fig. 1).

ATF6α is a type II ER transmembrane protein with a cytosolic cAMP-responsive element-binding protein (CREB)/ATF basic leucine zipper (bZIP) domain. Upon ER stress, ATF6α translocates to the Golgi apparatus and is cleaved by serine protease site-1 (S1P) and metalloprotease site-2 (S2P), leading to the production of an active transcription factor (pATF6α) [58–60] (Fig. 1). Then, pATF6α translocates to the nucleus, where it activates the expression of genes associated with protein folding and protein degradation [25, 61]. In addition, in response to ER stress, several cAMP-responsive element-binding proteins, including cAMP-responsive element-binding protein H (CREBH or CREB3L3), CREB3, CREB3L1, CREB3L2, and CREB, are activated similarly to ATF6α [62–64].

In addition to the canonical sensors mentioned above, Ca2^+^ is also an important player that transduces UPR signalling. Canonically, initiation of ER stress/the UPR is associated with a reduction in ER Ca^2+^ levels [65]. The reduction in ER Ca^2+^ enhances the UPR by the following mechanisms: (1) inhibition of the activity of several protein folding enzymes [66] and (2) impairment of the folding capacity by binding chaperones [66]. For example, Ca2^+^ binds to the chaperone calreticulin and activates it, leading to a reduction in misfolded proteins in the ER and thereby alleviating the UPR [67]. Mechanically, the Ca2^+^-binding chaperone calreticulin interacts with protein disulfide isomerase (PDI) and ERp57, two important protein folding enzymes that promote the proper folding of synthesized proteins, and activates them, thereby reducing the accumulation of misfolded proteins in the ER [68]. These studies demonstrated that Ca^2+^ plays an important role in the quality control and proper folding of newly synthesized proteins. Therefore, ER Ca^2+^ imbalance can greatly impact folding capacity and induce ER stress-mediated apoptosis. In multiple myeloma (MM), inhibition of TRPV1, which is involved in the regulation of calcium signalling, induces the accumulation of mitochondrial calcium and decreases the level of HSP70 induced by bortezomib, thereby impairing protein folding capacity and sensitizing MM cells to bortezomib [69].

## The source of the UPR

Due to cell-intrinsic factors, including the hyperactivation of oncogenes and inactivation of tumour suppressors, as well as the conditions of the tumour microenvironment, such as hypoxia and nutrient deprivation, cancer cells exhibit higher ER stress than normal cells [70]. Cell-intrinsic factors that trigger the UPR have been extensively reviewed elsewhere [70, 71]. In this section, we mainly focus on the impact of hypoxia on the UPR.

When tumour cells encounter a hostile environment, protein-folding homeostasis is disrupted, leading to activation of the UPR. All three UPR branches are activated by hypoxia, and the multiple mechanisms by which hypoxia activates the UPR have been identified. First, the formation of disulfide bonds during posttranslational folding or isomerization in the ER is oxygen-dependent [72]. Thus, hypoxia inhibits disulfide bond formation, leading to the accumulation of misfolded proteins in the ER [72]. Second, hypoxia promotes the expression of many components of the UPR, such as BIP [73, 74] and CHOP [75], through hypoxia-inducible factors. Third, hypoxia reduces the content of desaturated lipids and thereby limits the expansion of the ER, thus disrupting ER homeostasis [76, 77]. However, hypoxia can also increase the expression of ERO1α, an oxidoreductase involved in disulfide bond formation and protein folding in the endoplasmic reticulum, to restore ER homeostasis [78]. This phenomenon creates a feedback mechanism that contributes to the restoration of ER homeostasis. While writing this review, a detailed summary of hypoxia in relation to the unfolded protein response was published elsewhere [79, 80]. These findings contribute to our understanding of the well-established role of hypoxia in UPR activation.

### Role of the UPR in cancer

A moderate increase in UPR signalling can promote cell survival, whereas an excessive and prolonged UPR drives cells into apoptosis. This phenomenon applies to both normal and cancer cells because UPR activation-induced cell death pathways are integrated in at least some tumour cells. Thus, it is not surprising that the UPR could induce either the survival or apoptosis of cancer cells.

### The PERK-eIF2α pathway in cancer

The PERK-mediated UPR induces either survival or apoptosis in response to ER stress. Thus, the UPR may promote or inhibit malignant transformation [81], depending on the context. PERK deficiency has been shown to inhibit tumour progression [82], while activation of PERK signalling contributes to Myc-mediated malignant transformation by inducing autophagy [8, 83, 84]. In contrast, certain studies substantiate the fact that inhibition of PERK signalling promotes cell transformation [85]. For example, deletion of CHOP, a gene downstream of PERK signalling, has been reported to promote tumorigenesis in a Kras^G12V^-induced mouse model of lung cancer, indicating that CHOP has a tumour-suppressive role [86]. Moreover, PERK has been reported to be a haploinsufficient tumour suppressor in melanoma [85]. Thus, PERK is thought to be a tumour suppressor or proadaptive tumour promoter based on gene dose.

### The IRE1α-XBP1 pathway in cancer

IRE1α mutations have been found to be frequent in human cancers [87, 88]. Mounting evidence suggests that the IRE1α-mediated UPR branch could promote tumour development in glioblastoma by increasing the expression of genes associated with inflammation and angiogenesis, while loss-of-function mutations in the IRE1α signalling pathway increased the expression of matrix proteins, leading to inhibition of tumour invasion [89–93]. In addition, XBP1 inhibits lipid metabolism and antigen presentation in dendritic cells, leading to the suppression of antitumour immunity [94]. Studies have also shown that an increase in XBP1 expression can promote triple-negative breast cancer development by upregulating the expression of HIF-1α [95]. In contrast, in mouse models of intestinal cancer, loss of XBP1 function contributes to tumour incidence [89]. In multiple myeloma, loss-of-function mutations in IRE1α and XBP1 play pro-oncogenic roles [96], suggesting that the IRE1α-XBP1 pathway acts as a tumour suppressor in some tumours.

### The ATF6α signalling pathway in cancer

The role of ATF6α in cancer is poorly understood. Current evidence suggests that ATF6α is associated with hepatocellular carcinoma, bladder cancer, non-small cell lung cancer (NSCLC) and prostate cancer [97–100]. ATF6α functions as a transcription factor, which may be associated with the regulation of tumour cell dormancy [101]. Collectively, existing evidence indicates that ATF6α may help dormant cells adapt to chemotherapy and nutritional stress, although the underlying mechanism remains unclear.

## The UPR and chemotherapy resistance

Chemotherapy is an additional extrinsic challenge. In response to chemotherapeutic drugs, tumour cells have evolved a variety of mechanisms of drug resistance to adapt to this challenge. Multiple mechanisms associated with chemotherapy resistance have been identified, such as (i) a reduction in drug accumulation; (ii) enhancement of DNA damage repair (DDR); (iii) insensitivity of drug-induced cell death; (iv) induction of drug inactivation; and (v) induction of autophagy. Emerging evidence shows that the UPR is closely associated with the above mechanisms.

### The UPR and the reduction of drug accumulation

Drugs can enter tumour cells through diffusion, active transport, and endocytosis [102–104]. However, tumour cells limit this entry by increasing drug efflux or reducing drug uptake. The proteins responsible for drug efflux are named ATP-binding cassette (ABC) transporters; these transporters include multidrug resistance protein 1 (MDR1), MDR-associated protein 1 (MRP1), and breast cancer resistance protein (BCRP) [105–108]. In addition, downregulated expression or loss-of-function mutation of cell surface uptake transporters can reduce drug uptake. For example, downregulated expression or mutation of folate carriers reduced their drug affinity in osteosarcoma [109, 110].

**Fig. 2:**
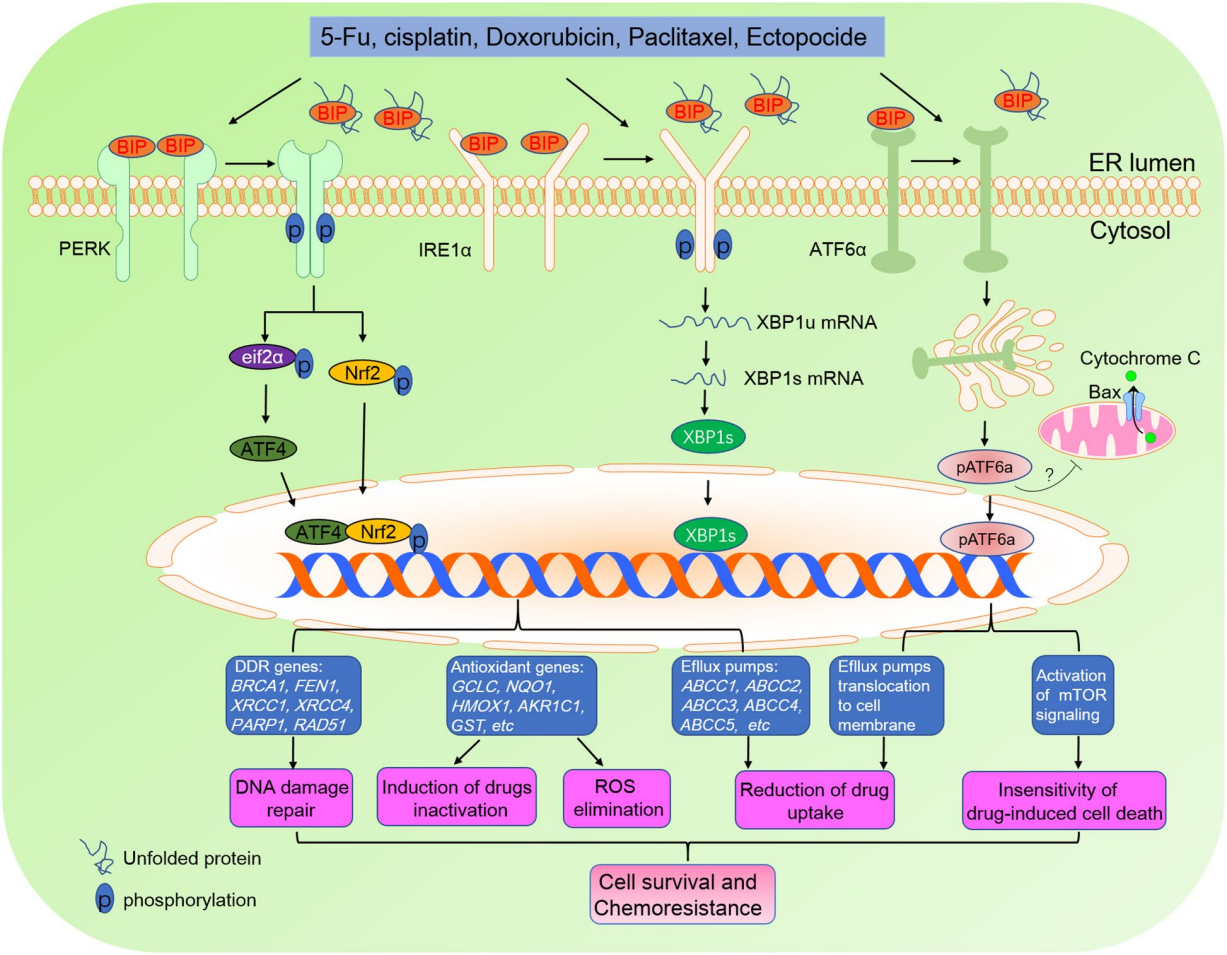
The UPR and chemotherapy resistance. UPR promotes chemotherapy resistance of tumor cells by increasing the expression of antioxidant genes, efflux pumps genes and DNA damage response (DDR) genes. In response to chemotherapeutic drugs, PERK directly phosphorylates NRF2, which increases the expression of antioxidant genes. The upregulation of antioxidant genes eliminates cellular ROS induced by chemotherapeutic drugs to promote cell survival. Certain antioxidant genes can also catalyze the conjugation of glutathione with chemotherapeutic drugs and lead to inactivation of chemotherapeutic drugs. In addition, activation of PERK can increase the expression of ATF4. ATF4 is closely associated with the expression of DDR and efflux pump genes. In response to chemotherapeutic drugs, the IRE1α-XBP1 pathway is also involved in the upregulation of a number of DDR genes. The third branch of UPR, ATF6, is associated with efflux pump translocation, anti-apoptosis and activation of mTOR signalling. Thus, activation of UPR is thought to  be crucial for chemotherapy resistance

Many studies have demonstrated that the UPR promotes drug efflux by upregulating the expression of ABC transporters. A recent study showed that the ATF4-mediated stress response–like transcriptional program is activated by daunorubicin, induces the expression of MDR1, and increases drug efflux [111], which leads to resistance to chemotherapeutic drugs (Fig. 2). Similarly, studies have also demonstrated that the 5-fluorouracil-activated IRE1α-XBP1 pathway upregulates the expression of ABC transporters and increases drug efflux in colon cancer cells [112]. In addition to the IRE1α-XBP1 pathway, the upregulation of GRP78 expression can also increase the efflux activity of ABC transporters, thereby conferring resistance to chemotherapeutic drugs in pancreatic cancer cells [113]. Additionally, in colorectal cancer, ATF6 is activated by TAM-secreted C–C motif chemokine ligand 17 (CCL17) and CCL22 via membrane receptor CCR4, leading to the upregulation of GRP78 expression [114]. Then, GRP78 interacts with MRP1 and promotes its translocation to the cell membrane, causing TAM-induced 5-FU efflux [114] (Fig. 2).

### The UPR and the increase in DNA damage repair

The antitumour activity of most drugs depends on the induction of DNA damage. For example, platinum-based drugs interact with DNA and form DNA inter- and intrastrand crosslinks, which leads to cancer cell apoptosis [115]. Anthracyclines, including adriamycin and daunorubicin, induce DNA damage in the following ways: (1) suppression of DNA replication and RNA synthesis by inhibiting DNA topoisomerase [116, 117]; (2) embedding of the DNA double strand and formation of DNA intrastrand crosslinks [118]; and (3) induction of ROS by Fe^3+^ chelation [119].

Many studies have demonstrated that DDR is associated with chemotherapy resistance. For instance, decreased expression of DNA topoisomerase I enhances the resistance of cancer cells to camptothecin [120]. Similarly, loss-of-function mutation of DNA topoisomerase-II, a target of doxorubicin and etoposide, has been reported in resistant cancer cell lines [121]. Furthermore, high expression of ERCC1, an important member of the nucleotide excision repair pathway, implies a poor response to chemotherapeutic drugs in many tumours, including NSCLC, gastric cancer and ovarian cancer [122, 123]. In particular, testicular cancer tissues have a very low ERCC1 level and are very sensitive to cisplatin [124]. A previous study also showed that overexpression of chromosomal instability (CIN) genes, which are involved in the maintenance of genomic integrity, is correlated with a poor prognosis for tumour patients [125].

An increasing body of evidence suggests that UPR sensors or downstream transcription factors are closely associated with DDR. For example, mammalian IRE1α-XBP1 has been identified to play an important role in the nonhomologous end-joining (NHEJ) repair pathway through the regulation of H4 acetylation [126]. Similar studies have shown that XBP1s in human hepatic cells directly upregulates the transcription of multiple DDR genes [127] (Fig. 2). Therefore, knockdown of XBP1s results in the formation and increase in γH2AX foci, downregulation of MRE11–RAD50–NBS1 (MRN) expression, and a decrease in ATM phosphorylation [128].

PERK-eIF2α-ATF4 is another UPR branch involved in DDR. Recent studies have shown that decreased expression of PERK in human breast cancer cells is associated with increased global phosphorylation of ATM and increased phosphorylation of its downstream effector CHK2, a cell cycle checkpoint kinase associated with DNA damage repair [82, 129] (Fig. 2). PERK-deficient tumour cells exhibit increased oxidative DNA damage, which leads to G2/M cell cycle checkpoint activation [82]. Moreover, PERK, IRE1α and ATF6α interact with DNA damage proteins (e.g., ATM, ATR, p53, p21, CHK1 and CHK2) to promote DDR in response to genotoxic stress, which enhances resistance to chemotherapeutic drugs [82, 128, 130–135]. Similarly, ATF4 increases the expression of cleavable topoisomerase complexes, leading to drug resistance to a variety of DNA-interactive agents [136–138].

### The UPR and insensitivity to drug-induced cell death

Mounting evidence indicates that cancer cells are usually "addicted" to a few antiapoptotic proteins and decrease the expression of proapoptotic proteins for their survival, thereby leading to resistance to chemotherapeutic drugs. These studies provide a strong theoretical basis for using these antiapoptotic and proapoptotic proteins as therapeutic targets. Mutations, amplifications, chromosomal translocations and overexpression of these antiapoptotic genes, including BCL‑2 family members, inhibitor of apoptosis proteins (IAPs), and the cellular FLICE-inhibitory protein (FLIP), are usually associated with chemotherapy resistance [139]. Antiapoptotic Bcl-2 family inhibitors have made substantial progress as anticancer therapies. UPR sensors, such as ATF4, upregulate the expression of the antiapoptotic genes BCL-2 and FLIP and protect tumour cells against apoptosis in response to drug-induced stress, leading to tumour cell resistance to chemotherapeutic drugs [139]. UPR sensors can also mediate the inactivation or downregulated expression of pro-apoptosis proteins and thus prevent tumour cells from undergoing chemotherapeutic drug-induced apoptosis. For instance, studies have demonstrated that GRP78 inhibits the pro-apoptosis proteins Bax (Bcl2-associated X protein), BIK (Bcl2-interacting killer) and caspase-7 activation [140–145], thereby reducing chemotherapeutic drug-induced apoptosis. In relapsed/refractory osteosarcoma (OS), ATF6α cleavage and activity were enhanced in OS cells compared to normal osteoblasts, and knockdown of ATF6α expression sensitized OS cells to chemotherapeutic drugs and induced cell death by activating Bax [146] (Fig. 2). In addition, UPR sensors can couple with other UPR sensors to promote tumour resistance to chemotherapeutic drugs. For example, functional coupling of GRP78 overexpression and PERK activation enhances dormant tumour cell resistance to chemotherapeutic drug-induced apoptosis [147]. Interestingly, GRP78 can interact with the tumour suppressor BRCA1 and inhibit apoptosis of ovarian and breast cancer cells [148]. Accordingly, silencing GRP78 increases ovarian and breast cancer cell sensitivity to chemotherapeutic drugs [148].

Moreover, UPR-mediated upregulation of antioxidant gene expression and the elimination of ROS may, at least in part, account for tumour cell resistance to chemotherapeutic drug-induced apoptosis. In response to chemotherapeutic drugs, PERK directly phosphorylates the transcription factor NRF2, a transcription factor that regulates the expression of antioxidant genes, leading to the elimination of drug-induced ROS and inhibition of cancer cell apoptosis [6, 149].

### The UPR and the induction of drug inactivation

Platinum drugs, including cisplatin, carboplatin, and oxaliplatin, can be covalently linked with thiol glutathione, which leads to their inactivation, decreases the availability of the native drug to bind its target, and promotes drug efflux by ABC transporters [130, 150]. Accordingly, high levels of glutathione have been reported to play a critical role in resistance to platinum drugs.

In response to chemotherapeutic drugs, the expression of ATF4 and NRF2, two downstream transcription factors of PERK, is upregulated, leading to increased expression of antioxidant genes, such as glutathione S transferase (GST) and glutamate-cysteine ligase catalytic subunit (GCLC) [2, 151, 152] (Fig. 2). Subsequently, GST conjugates hydrophobic electrophiles of drugs with glutathione, leading to inactivation of multiple chemotherapeutic drugs [130, 153]. Accordingly, targeting GST may be an effective therapeutic strategy against tumours.

### The UPR and the induction of autophagy

Autophagy is a cellular degradation process in which long-lived cellular proteins and organelles are encapsulated in double-membrane vesicles termed autophagosomes and degraded by lysosomal hydrolases [154]. Under normal conditions, autophagy occurs at basal levels and promotes the upkeep of cytoplasmic components. Under nutrient deprivation, oxidative stress, hypoxia and ER stress, autophagy can be significantly induced [155]. For example, under ER stress, the assembly of pre-autophagosomal structures is promoted and increases ATG1 kinase activity, leading to the induction of autophagy [156]. Many studies have demonstrated that PERK-regulated autophagy promotes tumour growth and contributes to the chemotherapeutic resistance of tumour cells. For example, PERK-mediated autophagy can enhance c-Myc-induced transformation and tumour growth [83]. When breast cancer cells are exposed to bortezomib, ATF4 is induced and triggers autophagy, which promotes breast cancer cell survival [7]. In response to chemotherapeutic drugs, the PERK-eIF2α pathway is activated and induces autophagy, which promotes drug resistance in human osteosarcoma [157]. In melanoma, PERK-mediated autophagy enhanced resistance to BRAF and MEK inhibitors through the PERK-ERK-ATF4 axis [158]. In this study, PERK phosphorylated ERK. Subsequently, ERK phosphorylated ATF4, which induced cytoprotective autophagy, leading to melanoma cell survival. Therefore, targeting PERK or ERK is a promising therapeutic strategy for overcoming resistance to BRAF and MEK inhibitors [158]. Additionally, the ATF6-mediated UPR was also found to promote chemotherapy resistance by inducing autophagy [159]. In high-grade serous ovarian cancer, ATF6 is activated by STAT3 and in turn induces the UPR to promote autophagy, thereby leading to cancer cell resistance to both cisplatin and paclitaxel treatment [159].

## The UPR and immunotherapy resistance

Cancer immunotherapies manipulate the immune system to recognize and attack cancer cells, and these therapies have revolutionized cancer therapy [160, 161]. However, immunotherapy resistance prevents most patients from benefiting from cancer immunotherapy. Recently, multiple mechanisms associated with immunotherapy resistance have been identified, including (1) impairment of T-cell recruitment; (2) deterioration of T-cell function; (3) suppression of antigen presentation; and (4) cancer cell stemness. Impairment of T-cell recruitment and deterioration of T-cell function in solid tumours are frequently associated with an immunosuppressive tumour microenvironment. An immunosuppressive tumour microenvironment is composed of all immune cells involved in the body’s immune response, such as CD4 + T cells, CD8 + T cells, dendritic cells, macrophages, and B cells, but the function of these immune cells is usually suppressed, which allows tumour cells to escape immune surveillance. Emerging evidence suggests that the UPR frequently participates in shaping an immunosuppressive tumour microenvironment and is closely associated with immunotherapy resistance.

### The UPR and the impairment of T-cell recruitment

Many tumours exhibit few T cells and a high number of immunosuppressive cells, including myeloid-derived suppressor cells, tumour-associated macrophages, and regulatory T cells, which exhibit immune-cold characteristics and lead to resistance to anti-PD1 therapy [162, 163]. In ovarian cancer, IRE1α-XBP1 signalling has been found to inhibit T-cell infiltration and IFNγ expression, thereby promoting tumour progression [164]. T-cell-specific XBP1 deletion increases T-cell infiltration, exhibits superior antitumour immunity and leads to inhibition of malignant progression and an increase in overall survival [164]. In patients with vitiligo, activation of IRE1α-XBP1 signalling increases CD8^+^ T-cell recruitment by upregulating CXCL16 expression, thereby leading to melanocyte-specific autoimmune responses and depigmentation of the skin [165]. Targeting the IRE1α-XBP1-CXCL16 axis could inhibit CD8^+^ T-cell infiltration and is thought to be a promising therapeutic strategy for treating vitiligo. In primary brain tumours, glioblastoma (GBM) is remarkably resistant to immunotherapy. Studies have demonstrated that XBP1-mediated expression of T-cell-suppressive checkpoints in myeloid cells, such as PD1, prevents the activation of tumour infiltrating T cells in GBM, thereby leading to resistance to anti-PD1 therapy [166]. These results suggest that XBP1 may suppress the activation of antitumour T cells in solid cancer by upregulating PD1 expression in myeloid cells. Therefore, targeting IRE1α-XBP1 signalling could help increase T-cell infiltration and enhance immunotherapy efficacy.

**Fig. 3:**
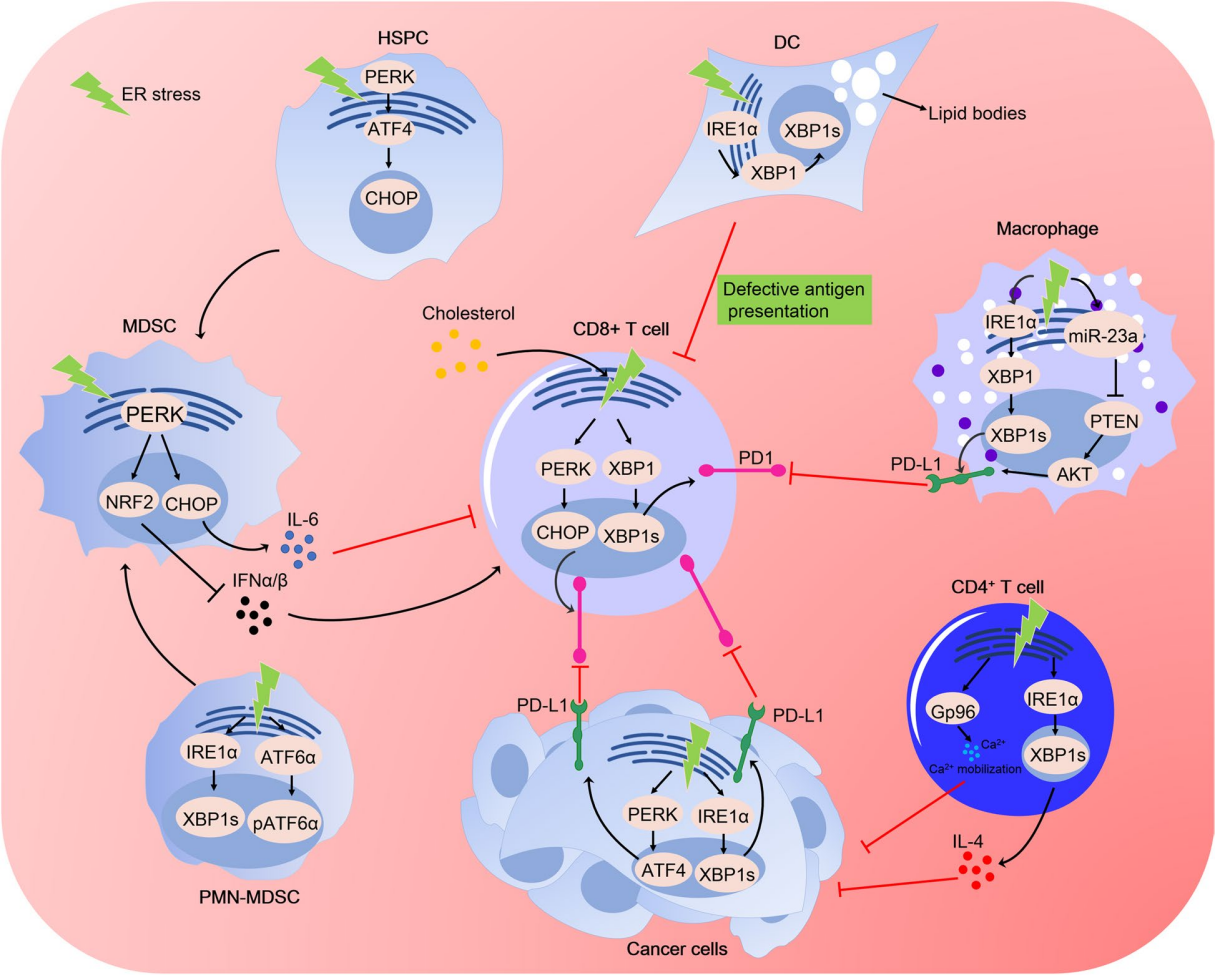
Effects of UPR signals on antitumor immunity. Nutrient starvation/excess or accumulation of ROS in tumor microenvironment leads to induction of ER stress in T cells and thus causes T cell exhaustion. High cholesterol levels in TME can activate PERK-eIF2α and IRE1α–XBP1 signaling in intratumoral T cells and induce the expression of PD1, which leads to the inhibition of CD8^+^ T cell antitumor immunity. Cancer cells undergoing ER stress activate PERK and IRE1α signals, which leads to upregulation of ATF4 and XBP1s. ATF4 and XBP1s directly upregulate the expression of PD-L1, triggering T cell exhaustion. Under hostile environmental condition, ER stress activates IRE1α and ATF6α and induces the transformation of HPSC into MDSCs. PERK can also induce the transformation of PMN-MDSCs into MDSCs, leading to immunosuppression. Among three UPR branches, activated PERK directly phosphorylates NRF2 and increases the expression of antioxidant genes, leading to limitation of ROS and maintenance of mitochondrial genome stability in MDSCs. This increase of mitochondrial genome stability inhibits the expression of IFN α/β and thereby suppresses T cell infiltration. Activated PERK also increases the expression of CHOP, leading to upregulation of IL-6 and thus promotes CD8^+^ T cell exhaustion. DCs are responsible for antigen processing and presentation and promote the transformation of T cells into CD8^+^T cells. ROS accumulation in DCs causes IRE1α-XBP1 activation, which drives uncontrolled lipid droplet formation and leads to the inhibition of antigen presentation of DCs. The hostile microenvironment can also activate IRE1α–XBP1 signaling and upregulate the expression of miR-23a in macrophages, leading to the increase of PD-L1 expression. Additionally, CD4^+^ T cells exploit IRE1α–XBP1 signaling to control Ca^2+^ mobilization and expression of IL4, which is necessary for the activation of CD8^+^ T cells

PERK-eIF2α signalling is also involved in the inhibition of CD8^+^ T-cell antitumour immunity. A previous study showed that activation of PERK-eIF2α signalling could inhibit CD8^+^ T-cell infiltration and promote tumour growth, while inhibition of PERK-eIF2α signalling decreased the number of infiltrating CD8^+^ T cells, increased tumour clearance, and enhanced the efficacy of anti-PD-1 therapy in sarcoma [167] (Fig. 3). Similarly, CHOP, a downstream sensor of PERK-eIF2α signalling, is widely thought to be a negative regulator of CD8^+^ T-cell antitumour immunity. An increase in CHOP expression has been shown in tumour-infiltrating CD8^+^ T cells and implies poor clinical outcomes in ovarian cancer patients [168]. CHOP deletion in T cells promotes spontaneous CD8^+^ T-cell antitumour immunity and increases the effectiveness of T-cell-based immunotherapy [169]. Thus, targeting the PERK-eIF2α pathway may be a promising strategy for overcoming immunotherapy tolerance and increasing the effectiveness of immunotherapy.

### The UPR and the deterioration of T-cell function

Deterioration of T-cell function refers to the loss of effector function and the expression of various immune checkpoint molecules in T cells, leading to a loss of tumour control. Multiple mechanisms associated with the deterioration of T-cell function have been identified, including (1) the expression of immune checkpoint molecules and their ligands; (2) the suppression of T-cell development and differentiation; and (3) the maintenance of the immunosuppressive function of myeloid-derived suppressor cells (MDSCs). An increasing number of studies show that the UPR is associated with the above mechanisms and causes tumour resistance to immunotherapy.

#### The UPR promotes the expression of immune checkpoint molecules and their ligands

Exposure to persistent antigens or inflammatory stimulation leads to the deterioration of T-cell function. Such deterioration is referred to as T-cell exhaustion, which is often associated with a loss of tumour control [21, 170–172]. Exhausted CD8^+^ T cells were characterized by high expression of immune checkpoint molecules, such as PD-1, LAG-3, TIM-3, 2B4, and CTLA-4 [173]. Many studies have shown that ER stress and chronic activation of the UPR lead to T-cell exhaustion [174–177]. In melanoma, high levels of cholesterol induce the expression of XBP1s, leading to upregulated expression of immune checkpoint molecules and inhibition of CD8^+^ T-cell antitumour immunity [178] (Fig. 3), while inhibition of XBP1 or a decrease in cholesterol could restore CD8^+^ T-cell antitumour immunity and reduce tumour progression [178]. Accordingly, reducing cholesterol or ER stress to restore T-cell function represents a new strategy to enhance the effectiveness of T-cell-based immunotherapy.

In addition to promoting the expression of immune checkpoint molecules on T cells, the UPR also promotes the expression of their ligands. Tumour-associated macrophages (TAMs) are abnormal myeloid cells that exist in the tumour microenvironment [179]. The relationship between TAMs and immune evasion has been well established. For example, TAM PD1 expression inhibits the phagocytic potency of macrophages against tumour cells. Accordingly, the PD1 inhibitor increases macrophage phagocytosis, reduces tumour growth, and prolongs the survival of mice in models of cancer in a macrophage-dependent fashion, which demonstrates that macrophages play a role in tumour evasion [180]. Recently, an increasing number of reports have demonstrated that the UPR is implicated in macrophage-associated immune evasion. In KSHV infection, the IRE1α-XBP1 pathway is activated and increases the expression of PD-L1 in KSHV-infected macrophages [181], leading to the loss of viral control (Fig. 3). In melanoma, activation of the IRE1α-XBP1 pathway upregulates the expression of macrophage PD-L1 and promotes tumour immune evasion [182] (Fig. 3). In addition, ER stress has been shown to upregulate PD-L1 expression in macrophages through the miR-23a–PTEN–AKT pathway, thereby promoting tumour progression [183] (Fig. 3). These studies show that ER stress-mediated PD-L1 expression in macrophages can inhibit T-cell function and promote tumour cell escape from antitumour immunity.

Although exhausted T cells lose their ability to kill tumour cells, this phenotype is reversible. The immune checkpoint blockade (PD1/PD-L1 immunotherapy) currently used in clinical practice can restore the antitumour function of T cells to a certain extent and improve the survival of tumour patients. Additionally, anti-PD1 combined with anti-HAVCR2, CTLA4, or TIGIT has been recently found to more effectively improve T-cell function, overcome resistance to anti-PD1 therapy, and eliminate tumour cells. These results show that high expression of multiple immune checkpoint molecules on T cells may be an important reason for immunotherapy resistance. Interestingly, XBP1 can simultaneously regulate the expression of multiple immune checkpoint molecules, such as PD1, LAG3, and HAVCR2, in some types of tumours [176], suggesting that XBP1 is a potential target for overcoming immunotherapy resistance.

#### The UPR regulates T-cell development and differentiation

In addition to CD8^+^ T cells, CD4^+^ T cells are also a part of the cancer immune cycle. CD4^+^ T cells play auxiliary roles for other cells of the immune system, especially antigen-presenting cells (APCs), such as macrophages, dendritic cells and B cells, and participate in the activation and maturation of these cells. CD4^+^ T cells have different subsets, such as Th1, Th2, Th17 and regulatory T cells. Growing evidence suggests that the UPR plays an important role in CD4^+^ T-cell differentiation and development and antitumour immunity. For example, in IRE1α knockout CD4^+^ T cells, CD4^+^ T cells cannot be activated and differentiated, leading to a decrease in IL4 production [184]. Additionally, a study showed that CD4^+^ T cells that are deficient in the ER stress chaperone GRP94 cannot be activated due to defective Ca^2+^ mobilization [185], leading to inhibition of tumour progression (Fig. 3). These studies suggest that UPR signalling is important for CD4^+^ T-cell differentiation and antitumour immunity. In another study, ATF4, a transcription factor of the PERK-eIF2α pathway, was shown to be essential for the CD4^+^ T-cell-mediated immune response [186]. Additionally, Yang et al. found that loss of ATF4 diminished Th1 effector function in high- and low-oxidizing environments. In this study, a moderate reduction in IL-17 production caused by loss of ATF4 was also observed, suggesting that ATF4 is involved in regulating Th17 cell development. In addition to ATF4, CHOP, another transcription factor of the PERK-eIF2α pathway, has also been implicated in the regulation of IL17 expression and Th17 cell differentiation. However, XBP1, a transcription factor of IRE1α-XBP1 signalling, inhibits the CD4^+^ T-cell-mediated immune response and promotes tumour progression [164]. Thus, moderate activation of PERK-eIF2α signalling combined with inhibition of IRE1α-XBP1 signalling in CD4^+^ T cells may promote the CD4^+^ T-cell-mediated immune response, allow cells to overcome immunotherapy resistance, and increase immunotherapy efficacy.

In addition to participating in CD4^+^ T-cell differentiation and development, the UPR also promotes CD8^+^ T-cell differentiation and the gain of effector function. Kamimura, D et al. found that XBP1 contributes to CD8^+^ T-cell differentiation during acute infection [187]. The ER stress chaperone GRP78 seems to be implicated in the regulation of CD8^+^ T-cell function. Studies have shown that GRP78 promotes the expression of granzyme B, an effector molecule, in CD8^+^ T cells [188], suggesting that the expression of GRP78 contributes to enhancing CD8^+^ T-cell cytotoxicity.

#### The UPR maintains the immunosuppressive function of MDSCs

MDSCs are marrow-derived heterogeneous cells that suppresses T-cell function [189–191]. PERK signalling has been demonstrated to be involved in the immunosuppressive function of MDSCs. For example, MDSCs expressing the ER stress sensor CHOP inhibited T-cell function and promoted tumour growth by upregulating IL-6 expression [192]. Accordingly, MDSCs lacking CHOP exhibited a decrease in immune regulatory functions and induction of antitumour responses, while IL-6 overexpression in CHOP-deficient MDSCs restored their immunosuppressive activity [192] (Fig. 3). These studies showed that IL-6 secreted by MDSCs was induced by CHOP and exerted immunosuppressive activity by suppressing T-cell function.

PERK signalling is also important for the maintenance of MDSCs. For example, activation of PERK signalling in MDSCs was found to be necessary for maintaining the MDSC population and immunosuppressive function [193] (Fig. 3). PERK deletion in MDSCs increased cytosolic mitochondrial DNA and activated the cGAS-Sting pathway, leading to the transformation of MDSCs into myeloid cells that activated CD8^+^ T-cell-mediated antitumour immunity [193] (Fig. 3). PERK signalling is also associated with the development of MDSCs [194] (Fig. 3). In mouse and human haematopoietic stem/progenitor cells (HSPCs), pharmacological and genetic inhibition of PERK signalling has been demonstrated to suppress the transformation of myeloid progeny cells into MDSCs [194]. These studies showed that PERK signalling plays an important role in the development, maintenance and regulation of MDSCs, suggesting that PERK signalling is a potential target of ICT.

Moreover, the IRE1α and ATF6α pathways were also involved in the maintenance of MDSCs. Tumour-bearing mouse models have shown that activation of the IRE1α and ATF6α pathways induces the development of polymorphonuclear MDSCs (PMNMDSCs) and promotes the immunosuppressive activity of PMNMDSCs (Fig. 3), thus leading to inhibition of the tumour-specific immune response and an increase in tumour progression [195]. These studies showed that the IRE1α and ATF6α pathways are important for MDSC-mediated immunosuppression, suggesting that targeting the IRE1α and ATF6α pathways is a promising therapeutic strategy for increasing the efficacy of ICT.

### The UPR and the impairment of antigen presentation

Antigen presentation is mediated by major histocompatibility complex (MHC) class I and MHC class II molecules and is essential for T-cell-dependent immune responses. Almost all cells express endogenous antigenic peptide-loaded MHC class I molecules on the surface, which are presented to cytotoxic CD8^+^ T cells and lead to CD8^+^ T-cell recognition of tumour cells and induction of the T-cell immune response. However, low expression of MHC class I molecules in tumour cells leads to the loss of efficient MHC class I-mediated antigen presentation and thereby promotes tumour cell evasion of immune surveillance and inhibits CD8^+^ T-cell infiltration [196–198]. Professional antigen-presenting cells (APCs), including dendritic cells, macrophages and thymic epithelial cells, constitutively express antigenic peptide-loaded MHC class II molecules (peptide-MHC class II) on the surface, which are presented to antigen-specific CD4^+^ T cells and lead to the activation of CD4^+^ T cells and the enhancement of T-cell-dependent anticancer immunity [197]. In tumour tissue, due to the immunosuppressive microenvironment, APCs cannot be effectively activated and effectively present antigens. Recent studies have shown that the UPR inhibits antigen presentation, leading to the suppression of T-cell-dependent anticancer immunity and tumour resistance to immunotherapy [199, 200]. The UPR inhibits antigen presentation by the following mechanisms: (1) the inhibition of antigen-presenting cell function and (2) the low expression of antigen processing and presentation genes.

#### The UPR inhibits the function of APCs

Conventional dendritic cells (cDCs) represent a diverse group of specialized antigen-presenting cells (APCs) that promote antitumour adaptive immunity by presenting antigens to T cells. Dendritic cells (DCs) are needed to initiate and sustain T-cell-dependent anticancer immunity [201]. Tumours often evade immune control by reducing normal DC function. A previous study showed that the UPR was involved in the inhibition of DC function. For example, constitutive activation of XBP1 in tumour-associated DCs (tDCs), driven by lipid peroxidation byproducts, has been found to induce a triglyceride biosynthetic program, which leads to abnormal lipid accumulation and subsequent inhibition of the capacity of tDCs to support antitumour T cells [94] (Fig. 3). DC-specific XBP1 deletion or selective nanoparticle-mediated XBP1 silencing in tDCs led to the restoration of immunostimulatory activity, which evoked protective type 1 antitumour responses and extended survival [94]. This study showed that targeting the XBP1-mediated ER stress response could significantly inhibit tumour growth and enhance anticancer immunity, thus providing a unique approach to cancer immunotherapy.

In addition to dendritic cells, the UPR also inhibits the antigen processing and presentation of macrophages by promoting a shift in M1–M2 polarization [202]. In melanoma, IREIα-XBP1 signalling is observed to promote M2 macrophage polarization, including the upregulation of interleukin 6 (IL-6), IL-23, and arginase 1 expression, leading to tumour immunosuppression [182]. Similarly, another group found that lipid-induced IRE1-XBP1 signalling increases the expression of Arginase1 and MRC1-associated M2 macrophages and facilitates M2 polarization [203]. In addition, in a mouse lung cancer model, XBP1 was found to upregulate the expression of IL10, TGFβ, and Arginase1 associated with M2 macrophages, downregulate the expression of IL-12, TNF-α, and iNOS associated with M1 macrophages, facilitate M2 polarization, decrease M2 macrophages in the tumour region, and enhance T-cell infiltration, thereby improving the efficacy of anti-PD1 therapy [204]. In addition to IRE1-XBP1 signalling, PERK-eif2α signalling has also been found to be involved in M2 macrophage polarization. CHOP, a transcription factor of the PERK-eif2α pathway, increased M2 macrophage production in a mouse model of bleomycin-induced pulmonary fibrosis, suggesting that CHOP inhibits antigen processing and presentation [205]. In addition, induction of PERK signalling in macrophages promotes immunosuppressive M2 activation and proliferation and inhibits the efficacy of anti-PD1 therapy in melanoma [206]. Although these studies reveal that the PERK-eif2α and IRE1-XBP1 branches of the UPR are promising therapeutic targets for sustaining host antitumour immunity, the overall picture is far from complete. In the future, we need to confirm which UPR branch in tumours is crucial for antigen presentation to guide targeted therapy combinations with immunotherapy.

#### The UPR inhibits the expression of antigen processing and presentation genes

The presentation of intracellular antigens by MHC-I is a complex process. First, antigens are primarily processed by the proteasome, which generates sources of peptides for MHC-I loading [207]. Then, these peptides are imported into the ER by the transporter associated with antigen processing (TAP). Subsequently, the peptide-loading complex (PLC), which is composed of MHC-I heavy chain and b2 microglobulin, TAP, tapasin, calreticulin and ERp57, promotes the binding of newly synthesized MHC-I molecules to these peptides and thereby forms peptide–MHC-I complexes [207]. Finally, peptide–MHC-I complexes are released from the ER and transported via the Golgi to the plasma membrane for antigen presentation to CD8^+^ T cells [207].

Notably, IRE1α is involved in the regulation of several members of the MHC-I antigen presentation pathway. Guttman et al. found that DCs process internalized protein antigens into antigen-derived peptides, enter the ER and masquerade as unfolded proteins, thereby leading to the activation of IRE1α [199]. IRE1α activation promotes MHC-I heavy-chain mRNA degradation by regulating IRE1α-dependent decay (RIDD) and attenuating antigen cross-presentation [199]. In tumour-bearing mice, IRE1α inhibition enhanced MHC-I expression on tumour-infiltrating DCs and increased CD8^+^ T-cell infiltration and activation [199]. IRE1α inhibition synergized with anti-PD-L1 therapy to inhibit tumour progression and enhance the efficacy of immunotherapy [199]. At the cellular level, activation of IRE1α upregulates the expression of miR-346. The increase in miR-346 expression downregulates the expression of its target genes, including MHC I and TAP, leading to inhibition of MHC I-mediated antigen presentation [189]. In mice infected with *Toxoplasma gondii*, infection led to specific activation of the IRE1α pathway of the cDC1 subset, while IRE1α promoted MHC I antigen presentation of secreted parasite antigens [190]. This evidence indicates that IRE1α is needed to inhibit the gene expression of key members of the MHC-I antigen presentation pathway, suggesting that targeting IRE1 is a promising strategy for cancer immunotherapy.

### The UPR and cancer cell stemness

Cancer stem cells (CSCs) refer to a small subpopulation of cancer cells that can self-renew, recapitulate the heterogeneity of original tumours, and differentiate into the whole bulk of a new tumour. Cancer stem cells have been shown to increase resistance to cancer immunotherapy in many studies [191, 208]. For example, cancer stem cells suppress CD8 + T-cell infiltration and promote the recruitment of type 2 macrophages (M2), leading to systemic immunosuppression and subsequent immunotherapy resistance [209]. In a skin cancer model for squamous cell carcinoma (SCC), transforming growth factor β (TGF-β)-responsive tumour-initiating stem cells promoted skin cancer resistance to adoptive cytotoxic T-cell transfer (ACT)-based immunotherapy [191]. Notably, the UPR is implicated in the regulation of cancer stem cells. In ovarian cancer, FOXK2, as a highly expressed stemness-specific transcription factor, increased stemness features and tumour initiation capacity by directly activating the IRE1α-XBP1 pathway, while genetic or pharmacological blockade of this pathway inhibited ovarian CSCs [210]. In breast cancer, ER-associated protein p97 inhibition increased the expression of multiple stemness and pluripotency regulators, including C/EBPδ, c-MYC, SOX2, and SKP2, by activating the PERK-eif2α pathway, leading to an increase in CSCs [211]. In GBM, PERK signalling was also found to promote the expression of the stemness regulator SOX2 and increase GBM cell stemness [212]. Moreover, the epithelial-to-mesenchymal transition (EMT), a phenotype of CSCs, is thought to promote both tumour progression and drug resistance. A study found that EMT gene expression correlates strongly with that of PERK-eIF2α genes, suggesting that the PERK-eif2α pathway is involved in the regulation of CSCs [213]. These studies revealed that the UPR plays an important role in CSCs and is a promising therapeutic target for cancer treatment. Many other components of the UPR that are involved in the regulation of CSCs have been described and reviewed elsewhere. These findings contribute to our understanding of the relationship between the UPR and CSCs.

## Targeting the UPR to overcome chemotherapy resistance

Given that cancer cells exhibit elevated levels of ER stress, these malignant cells could be dependent on resistance to ER stress for cell survival. Thus, targeting the UPR may be a promising strategy for cancer treatment [214, 215].

### Targeting the IRE1α-XBP1 pathway

As acute ER stress promotes tumour cell apoptosis, agents that elevate ER stress promote apoptosis in cancer cells (Table 1). The ER stress-generating agent bortezomib, the first proteasome inhibitor approved by the US Food and Drug Administration (FDA) for cancer treatment, functions as an XBP1s activator to treat multiple myeloma [216]. Studies have shown that XBP1 (or XBP1s) levels are positively correlated with bortezomib efficacy in patients with multiple myeloma [217], suggesting that XBP1s can act as a predictive marker of treatment outcomes.

**Table 1:** UPR-targeting agents in cancer

Target	Agents	Cancer types	Functions	Refs
IRE1α	Bortezomib	Multiple myeloma	Activates XBP1 mRNA splicing	[216]
	MKC-3946	Multiple myeloma	Inhibits XBP1 mRNA splicing	[218]
	4μ8C	Multiple myeloma	Inhibits XBP1 mRNA splicing	[219]
	STF-083010	Multiple myeloma	Inhibits XBP1 mRNA splicing	[220]
	APY29	Various cancers	Inhibits IRE1α kinase activity	[221]
	Toyocamycin	Various cancers	Inhibits XBP1 mRNA splicing	[222]
	Sunitinib	Various cancers	Inhibits IRE2α kinase activity	[223, 224]
	B-I09	Multiple myeloma	Inhibits XBP1 mRNA splicing	[225, 226]
PERK	GSK2606414	Various cancers	Inhibits PERK and eIF2α phosphorylation, ATF4 translation and CHOP expression	[227]
	GSK2656157	Various cancers		[228]
HSP90	17-AAG	Various cancers	Activates UPR	[229, 230]
	Radicicol	Various cancers	Activates UPR	[229]
	AUY-922	Various cancers	Activates UPR	[231]
	IPI-504	Various cancers	•Inhibits the activation of transcription factors XBP1 and ATF6, and blocks the tunicamycin-induced eIF2α phosphorylation by PERK •Prevents GRP78 accumulation	[232]
GRP78	DHA	Various cancers	Suppresses GRP78 expression	[233]
	PAT-SM6	Multiple myeloma	Interacts with multiple BIP on cancer cell surface, and inhibits BIP activity	[234]
	Arctigenin	Various cancers	Specifically inhibits the transcriptional induction of BIP and GRP94 under glucose deprivation	[235]
VCP	ML240	Multiple myeloma	Inhibits ERAD pathway	[236]
	Eeyarestatin	Cervical cancer, non-small cell lung cancer	Inhibits ERAD pathway	[237]

*IRE1α* Inositol-requiring enzyme 1, *ATF4* Activating transcription factor 4, *eIF2α* Eukaryotic translation factor 2α, *PERK* Protein kinase RNA-like ER kinase, *HSP* Heat shock protein, *XBP1* X-box binding protein 1, *CHOP* C/EBP-homologous protein, *GRP78* Glucose-Regulated Protein, *ERAD* ER-associated degradation, *UPR* Unfolded protein response, *VCP* Valosin-containing protein

**Fig. 4:**
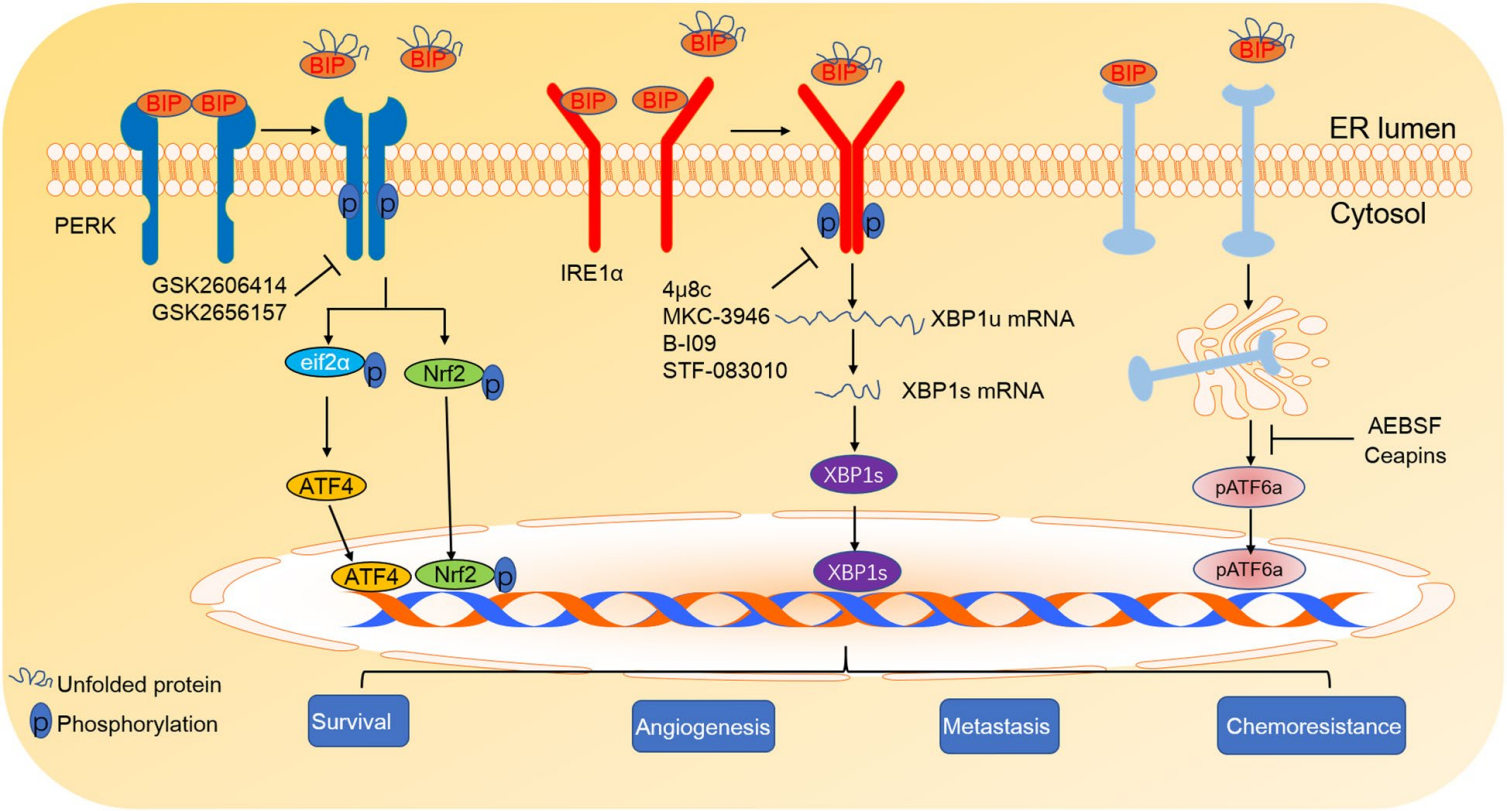
Therapeutic strategies to target UPR in tumors. Compounds, such as 4μ8c, MKC-3946 B-I09, and STF-083010 can directly inhibit IRE1α ribonuclease domain and prevent XBP1 RNA splicing. PERK inhibitors, such as GSK2606414 and GSK2656157, can directly inhibit activation of PERK and overcome chemotherapy resistance. ATF6 inhibitors, such as AEBSF and Ceapins, can directly inhibit the transactivation of ATF6. Treatment with these IRE1α, PERK, or ATF6 inhibitors can effectively reduce the hypoxia tolerance, angiogenesis, drug resistance and tumor metastasis

Inhibition of IRE1α-XBP1 signalling can attenuate cancer cell adaptation to ER stress and augment ER stress, which promotes tumour cell apoptosis; this method could be used as an anticancer strategy (Fig. 4). Recently, some new drugs have been developed to inhibit the IRE1α-XBP1 pathway and augment ER stress. IRE1α inhibitors targeting the catalytic core of the RNase domain or ATP-binding pocket of the kinase domain, such as sunitinib, APY29, toyocamycin, STF-083010, 4μ8C, MKC-3946 and B-I09 [218–221, 223–225, 238, 239], were identified by high-throughput screens (Table 1). Among these inhibitors, sunitinib and APY29 inhibit IRE1α kinase activity by interacting with the ATP-binding pocket of the kinase domain [238], while toyocamycin, STF-083010, 4μ8C, MKC-3946 and B-I09 inhibit IRE1α RNase activity by targeting the catalytic core of the RNase domain. MKC-3946 and STF-083010 have been demonstrated to inhibit tumour formation in multiple myeloma xenograft models [219, 226], while toyocamycin produced by an *Actinomycete* strain synergistically potentiated the therapeutic effectiveness of bortezomib by inducing the apoptosis of multiple myeloma cells at nanomolar concentrations [222]. Notably, although sunitinib and APY29 are thought to inhibit IRE1α kinase activity, some studies have demonstrated that they also activate IRE1α RNase activity in vitro [221, 224].

### Targeting the PERK-eIF2α pathway

Since the PERK-eIF2α pathway plays an important role in the drug resistance of cancer cells [81, 240], targeting the PERK-eIF2α pathway with chemotherapy is thought to be effective for cancer treatment and contributes to overcoming chemotherapy resistance (Fig. 4). At present, PERK-eIF2α signalling inhibitors have been designed and developed for cancer treatment. For example, GSK2606414, the first reported PERK inhibitor, inhibits the PERK-mediated UPR and increases the amount of misfolded proteins in the ER [227], resulting in cancer cell apoptosis (Table 1). GSK2606414 has demonstrated oral activity and decreased tumour growth in a xenograft model of pancreatic cancer [227]. GSK2656157, another PERK inhibitor, has been reported to have better inhibitory properties (Table 1) [228]. Studies have shown that GSK2656157 inhibits ER stress-induced PERK autophosphorylation and eIF2α phosphorylation in multiple cell lines [228]. The twice-daily dosing of GSK2656157 results in dose-dependent inhibition of multiple human tumour xenograft growth in mice. The antitumour activity of GSK2656157 may be attributed to alterations in amino acid metabolism and decreases in blood vessel density and vascular perfusion, which led to dose-dependent inhibition of the growth of multiple human tumour xenografts in mice [228].

### Targeting the ATF6α pathway

Although ATF6α is proposed to promote the dormancy of tumour cells and resistance to chemotherapeutic drugs, ATF6α inhibitors have rarely been developed. 4-(2-Aminoethyl) benzenesulfonyl fluoride (AEBSF), a serine protease inhibitor, has been found to prevent ER stress-induced cleavage of ATF6α and ATF6β (Fig. 4), resulting in the inhibition of transcriptional induction of ATF6 target genes [241]. Mechanically, AEBSF seems to directly prevent the cleavage of ATF6α and ATF6β by inhibiting Site-1 protease [241]. Using ER stress response (ERSE)-luciferase assays, Gallagher et al. discovered and developed Ceapins, a class of pyrazole amides that block ATF6α signalling in response to ER stress [242] (Fig. 4). Further study found that Ceapins are highly specific inhibitors of ATF6α signalling and do not affect signalling through the other branches of the UPR. Genome-wide CRISPR interference and proteomics studies revealed that the ABCD3 peroxisomal transporter interacts with ER-resident ATF6α in a Ceapin-dependent manner, leading to the inhibition of ATF6α cleavage [243].

### Targeting other components of the UPR

Other agents that inhibit UPR signalling include chaperone and ERAD pathway inhibitors. For example, chaperone HSP90 and GRP78 inhibitors include 17-AAG [229, 230], AUY-922 [231], IPI-504 [232], radicicol [229], docosahexaenoic acid (DHA) [233], PAT-SM6 [234] and arctigenin [235] (Table 1), which bind to the amino-terminal ATP-binding domain of their targets and lead to cell apoptosis. ERAD pathway inhibitors include ML240 [236] and Eeyarestatin [237] (Table 1), which are involved in the suppression of valosin-containing protein (VCP) ATPase.

## Targeting UPR to enhance the efficacy of immunotherapy

The UPR is closely associated with the antitumour activity of immune cells [244]. Many studies have shown that targeting the UPR in combination with ICT is an effective therapeutic strategy for tumour treatment.

### Targeting PERK-eif2α signalling

PERK-eif2α signalling plays an important role in T-cell exhaustion, the development and maintenance of MDSCs, and PD-L1 expression in tumour cells, suggesting that targeting PERK-eif2α signalling is a promising strategy for the treatment of cancers. GSK2606414, a PERK inhibitor, significantly increased CD8^+^ T-cell infiltration, inhibited tumour growth, and enhanced the efficacy of anti-PD1 immunotherapy [167]. Similar to GSK2606414, an ERO1α (a PERK axis target gene) inhibitor also promoted CD8^+^ T-cell immunity, controlled tumour progression, and improved the immunotherapy response in mouse tumour models [167]. In addition, GSK2606414 and AMG-44 (a potent and selective PERK inhibitor) have been reported to activate cGAS-sting signalling in MDSCs by inhibiting the NRF2-mediated antioxidant pathway, thereby impairing the immunosuppressive activity of tumour MDSCs. This impairment increases the infiltration of CD8^+^ T cells and delays tumour growth in B16 tumour-bearing mice [193]. Surface-localized GRP78 is also considered to be a potential target of immunotherapy [245, 246]. These studies suggest that targeting PERK-eif2α signalling enhances the efficacy of ICIs and could provide benefits for cancer patients.

### Targeting IRE1α-XBP1 signalling

An increasing number of studies have demonstrated that IRE1α-XBP1 signalling is associated with immunosuppression, suggesting that IRE1α-XBP1 signalling may be a potential target for immunotherapy. STF083010, an IRE1α RNase inhibitor, could impair PD1 expression in CD8^+^ T cells by inhibiting the transcriptional activity of XBP1 and enhancing the antitumour immunity of CD8^+^ T cells in mouse models of melanoma [176]. In a CARM1-expressing ovarian cancer model, the IRE1α inhibitor B-I09 increased the effectiveness of anti-PD1 therapy [225]. However, the underlying mechanism is still unclear. A reasonable explanation is that XBP1s may have promoted PD1 expression in a CARM1-expressing ovarian cancer model and thus increased the efficacy of anti-PD1 therapy. In addition, IRE1α-XBP1 signalling increased PD-L1 expression in TAMs and thus promoted melanoma growth [182], suggesting that targeting IRE1α-XBP1 signalling to TAMs combined with anti-PD-L1 therapy may be an effective strategy for the treatment of melanoma. These studies demonstrate that targeting IRE1α-XBP1 signalling may be a potential strategy to enhance the effectiveness of immunotherapy.

## Conclusions

When tumour cells encounter a hostile microenvironment, such as nutrient deprivation, oxygen limitation, oxidative stress or chemotherapeutic drugs, the protein-folding capacity of the ER is disturbed, leading to activation of the UPR. Over the past decade, although we have made great progress in understanding the role of the UPR in cancer, many questions remain. For example, UPR-induced cell death pathways are integrated into some tumour cells. How do cancer cells avoid cell death in response to chronic UPR activation? Preclinical studies have suggested that a therapeutic threshold exists for UPR inhibitors. How can the optimal dose of UPR inhibitors be determined in individual patients? Chemotherapy represents an additional extrinsic challenge that cancer cells have to face and to which they adapt in the case of resistance. The main problem is how to determine the window of opportunity to target the UPR, that is, when UPR-targeting agents should be given after chemotherapy.

ICT has revolutionized cancer management, but resistance to ICT is emerging as an urgent problem to be solved. Increasing evidence suggests that targeting UPR sensors or UPR-associated components could sensitize aggressive tumours to immunotherapy, but the availability of UPR targets is limited. Thus, larger prospective and preclinical studies and retrospective clinical trial analyses are needed to uncover potent UPR targets to overcome resistance to chemotherapeutic drugs and ICIs and prevent cancer progression and/or recurrence.

## Data Availability

Not applicable.

## Abbreviations

ER: Endoplasmic reticulum
UPR: Unfolded protein response
ICT: Immune checkpoint therapy
PERK: Protein kinase RNA-like endoplasmic reticulum kinase
IRE1: Inositol-requiring enzyme 1
ATF6: Activating transcription factor 6
ATF4: Activating transcription factor 4
BiP: Binding immunoglobulin protein
GADD34: Growth arrest and DNA damage-inducible protein 34
DC: Dendritic cell
XBP1: X-box binding protein 1
ERAD: ER-associated protein degradation
ROS: Respective oxygen species
TME: Tumor microenvironment
PD1: Programmed cell death protein 1
MDSC: Myeloid-derived suppressor cells
